# Effects of Encapsulated Cells on the Physical–Mechanical Properties and Microstructure of Gelatin Methacrylate Hydrogels

**DOI:** 10.3390/ijms20205061

**Published:** 2019-10-12

**Authors:** Srikumar Krishnamoorthy, Behnam Noorani, Changxue Xu

**Affiliations:** 1 Department of Industrial, Manufacturing, and Systems Engineering, Texas Tech University, Lubbock, TX 79409, USA; srikumar.krishnamoorthy@ttu.edu; 2 School of Pharmacy, Texas Tech University Health Sciences Center, Amarillo, TX 79106, USA; behnam.noorani@ttuhsc.edu

**Keywords:** gelatin methacrylate (GelMA), encapsulated cells, physical–mechanical properties, microstructure, tissue engineering

## Abstract

Gelatin methacrylate (GelMA) has been gaining popularity in recent years as a photo-crosslinkable biomaterial widely used in a variety of bioprinting and tissue engineering applications. Several studies have established the effects of process-based and material-based parameters on the physical–mechanical properties and microstructure of GelMA hydrogels. However, the effect of encapsulated cells on the physical–mechanical properties and microstructure of GelMA hydrogels has not been fully understood. In this study, 3T3 fibroblasts were encapsulated at different cell densities within the GelMA hydrogels and incubated over 96 h. The effects of encapsulated cells were investigated in terms of mechanical properties (tensile modulus and strength), physical properties (swelling and degradation), and microstructure (pore size). Cell viability was also evaluated to confirm that most cells were alive during the incubation. It was found that with an increase in cell density, the mechanical properties decreased, while the degradation and the pore size increased.

## 1. Introduction

The encapsulation of living cells inside biomaterials is an approach in tissue engineering that allows for the engineering of living tissues with structural and biochemical similarities to natural tissue [1]. Hydrogels are widely used due to important properties such as a high water content, biocompatibility, and their ability to mimic the microstructure of a cell’s natural extracellular matrix (ECM) [2,3]. Hydrogel physical–mechanical properties and microstructures are of great importance to cell attachment, viability, differentiation, and proliferation as well as the eventual functionality of the fabricated tissues. Specifically, hydrogel mechanical properties significantly affect the functionality of the fabricated tissue constructs [4]. Hydrogel physical properties mainly include swelling and degradation. Swelling represents the amount of excess water that can be held within a hydrogel, and this can directly impact the permeation of nutrients into the gel and the egestion of cellular waste products out of the gel, as well as the shape fidelity of the printed constructs [5]. The degradation can be described as the breakdown of the crosslinks or the polymer backbone of a hydrogel by hydrolysis due to various enzymes [6]. This enables the synthesis of natural ECM by encapsulated cells and eventual tissue growth. It is important for hydrogel degradation to be optimal in order to ensure tissue growth without having an adverse effect on the surrounding hydrogel material [7]. Moreover, nondegradable hydrogel crosslinks can be toxic to encapsulated cells and inhibit tissue formation [8]. Hydrogel microstructure, mainly pore size, is of great importance to the effective transfer of oxygen and nutrients to encapsulated cells [9]. These encapsulated cells are typically inhibited in their movement due to being surrounded by the hydrogel matrix and are reliant on the pores for the uptake of nutrients [10]. Pores of the hydrogels also provide spaces for cell attachment and proliferation [11].

Gelatin methacrylate (GelMA) is a hydrogel that has recently been gaining popularity in bioprinting and 3D tissue culture applications [12,13]. GelMA is synthesized when methacrylic anhydride reacts with the primary amine groups of gelatin at elevated temperatures to result in the addition of methacrylate groups onto the gelatin macromers. GelMA is a photo-crosslinkable derivative of gelatin, possessing similar biocompatible properties to gelatin due to their similar molecular structures [12]. Sutter et al. [14] described size exclusion chromatograms for both gelatin and GelMA with a high degree of functionalization and observed that the products had a retention time of 23.3 min, which corresponded to a similar molecular weight of around 89 kDa in both cases. The crosslinking of GelMA occurs when GelMA is exposed to UV radiation in the presence of a photoinitiator. Free radicals are generated by the photoinitiator upon the absorption of UV light, which subsequently polymerizes the GelMA [5,15]. GelMA has been used in various 3D bioprinting and tissue engineering applications, such as the fabrication of vascular networks [16] and the formation of micropatterns within cell-encapsulated GelMA hydrogels [5]. The critical factors that influence the properties of GelMA are the concentration [12,17], the degree of functionalization of gelatin [16,18], and the parameters of photo-crosslinking, such as UV intensity and exposure time [18,19]. Namely, Zhao et al. [12] reported that with an increase in the GelMA concentration, the mechanical strength increases, the swelling decreases, and the degradation decreases. Schuurman et al. [17] reported that with an increase in the GelMA concentration, the mechanical strength increases, and the swelling decreases. Chen et al. [16] reported that with an increase in the GelMA degree of functionalization, the pore size of the GelMA hydrogels decreases, while the compressive modulus increases. Colosi et al. [19] reported that the GelMA mechanical strength increases with an increase in the UV intensity and exposure time.

Although many studies have been reported regarding the effects of critical factors such as hydrogel concentration [12], the degree of functionalization of gelatin [16], UV intensity, and exposure time [18] on GelMA physical–mechanical properties and microstructures, the effect of encapsulated cells is still missing. When living cells are encapsulated within GelMA hydrogels, they release enzymes through their membrane. As these enzymes diffuse through the polymer network, the enzymes act as catalysts to cause hydrogel degradation, which can potentially affect GelMA properties. Hence, it is hypothesized that the encapsulated cells affect GelMA physical–mechanical properties as well as the microstructure. The objective of this study was to quantitatively investigate the effects of encapsulated cells on the physical–mechanical properties and microstructure of GelMA hydrogels. This paper is organized in the following manner: First, the experimental materials and methods are introduced in detail. Second, the GelMA degree of substitution is measured and discussed. Third, the effects of encapsulated cells on the mechanical and physical properties and microstructure of GelMA hydrogels are investigated, including tensile modulus and strength, degradation and swelling, and pore size. Finally, the major conclusions are listed, and future work is proposed.

## 2. Results and Discussion

### 2.1. Characterization of GelMA Functionalization

**Figure 1:**
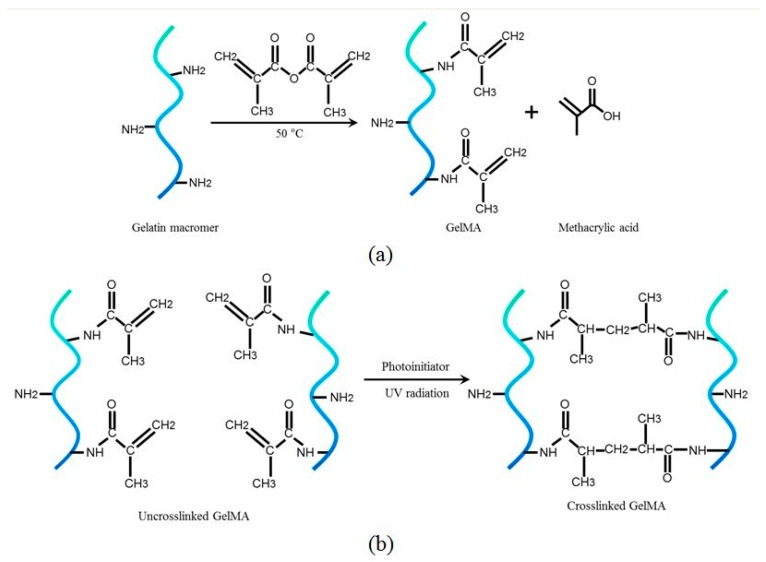
(**a**) Synthesis of gelatin methacrylate (GelMA) through the addition of methacrylamide groups onto the gelatin macromers and (**b**) the crosslinking of GelMA under UV radiation in the presence of a photoinitiator.

The synthesis of GelMA is shown in Figure 1a. Methacrylic anhydride reacts with the primary amine groups of gelatin at an elevated temperature of 50 °C, resulting in the addition of methacrylamide groups onto the gelatin macromers [5,15]. The crosslinking of GelMA occurs when GelMA is exposed to UV radiation in the presence of a photoinitiator. The photoinitiator generates free radicals upon the absorption of UV light, and GelMA is subsequently polymerized, as shown in Figure 1b [5,15]. Typically, not all of the amine groups on gelatin macromers are substituted with methacrylamide bonds. The degree of functionalization (DoF) is used to characterize the number of amine groups in the gelatin macromers substituted with methacrylic groups [20]. The DoF can be tuned by changing the amount of methacrylate anhydride added and the reaction parameters such as temperature and pH. In this section, two analyses were performed: ^1^H NMR spectroscopy was used to confirm the presence of methacrylamide groups grafted onto the gelatin macromers as part of the synthesis process, and the DoF of the GelMA was quantified using a primary amine-reactive fluorescent detection reagent.

**Figure 2:**
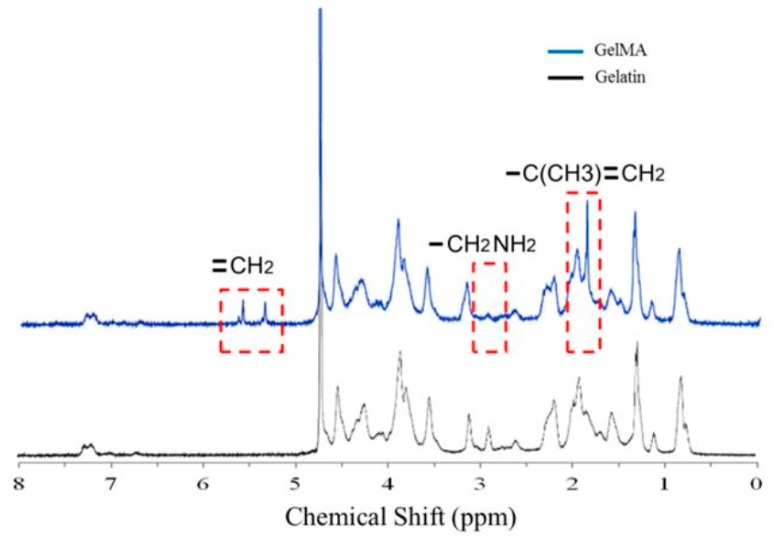
Here, 400-MHz ^1^H NMR spectra for unsubstituted gelatin and GelMA are shown. The peaks due to the added methacrylate were between 5 and 6 ppm and between 1.5 and 2 ppm. The peaks due to the lysine methylene from gelatin were between 2.5 and 3 ppm.

GelMA consists of methacrylamide bonds grafted onto gelatin macromers. The presence of methacrylamide in the gelatin hydrogel was quantified using ^1^H NMR spectroscopy. Samples of GelMA and gelatin were dissolved in deuterium oxide (D_2_O) at a concentration of 3% (*w/v*). The spectra were collected at 37 °C (to avoid gel formation of gelatin and GelMA) at a frequency of 400 MHz using a Bruker NMR Spectrometer (Bruker, Billerica, MA, USA). The spectrum in Figure 2 shows a complex fingerprint from amino acid residues in gelatin itself and the grafted methacrylamide moieties. The peaks between 5 and 6 ppm on the spectra were due to acrylic protons from the mectacrylate, and the peaks between 1.5 and 2 ppm were due to the methyl function from the methacrylate [21]. The peaks between 2.5 and 3 ppm were due to the lysine methylene from gelatin [21]. The graph confirmed the incorporation of the acrylamide double bonds at 5.3 and 5.6 ppm [22], and the double bonds were necessary for the photopolymerization of GelMA.

OPA (*o*-phthalaldehyde) is a primary amine-reactive fluorescent detection reagent that can be used as a protein/peptide assay reagent. The reaction of peptides and proteins to OPA has a linear relationship over a wide range of concentrations. GelMA solutions and gelatin solutions with different concentrations (0.002%, 0.01%, 0.05%, 0.1%, and 0.15% (*w/v*)) were prepared by dissolving them in Dulbecco’s phosphate-buffered saline (DPBS). These solutions were reacted with the reagent at a ratio of 2:1 (*v/v*) for 60 s. Subsequently, an excitation/emission of 340/455 nm was used by a microplate reader to measure the fluorescent intensity of the samples. The GelMA DoF can be calculated as follows:

$$DoF\;\left(Degree\;of\;Functionalization\right)=1-\left(\frac{\left(I_{GelMA}-I_{DPBS}\right)}{\left(I_{Gelatin}-I_{DPBS}\right)}\right)$$

where *I_GelMA_* is the fluorescent intensity of the GelMA sample, *I_Gel__atin_* is the fluorescent intensity of unsubstituted gelatin, and *I_DPBS_* is the fluorescent intensity of DPBS, as measured by the microplate reader. The calculated DoF for the GelMA used in this study was 75%. Generally, GelMA can be classified into three types depending on the DoF: GelMA with a low DoF has up to 40% amine groups substituted; GelMA with a high DoF has at least 65% amine groups substituted; and GelMA with a medium DoF has an intermediate percentage of substitution [17,21]. The GelMA DoF significantly affects the physical properties, such as the swelling ratio, stiffness, and mechanical strength. Typically, with an increase in the GelMA DoF, the elastic modulus increases and the degradation decreases. Hence, GelMA with a high DoF is preferred in various tissue engineering applications [23,24]. In this study, the GelMA DoF was 75%, which is considered to be a high DoF.

### 2.2. Mechanical Properties

The mechanical properties of hydrogels are commonly characterized by compression testing. However, compression testing does not lend itself well to yielding reproducible data, nor does it provide fracture toughness [25]. Moreover, for the biofabrication of vascular-like constructs for biomedical applications in tissue-engineered blood vessels [26,27,28], the mechanical properties related to resistance to tensile strain and tensile strength are extremely important [25,29]. Hence, the mechanical properties in this study were characterized in terms of tensile modulus and strength.

**Figure 3:**
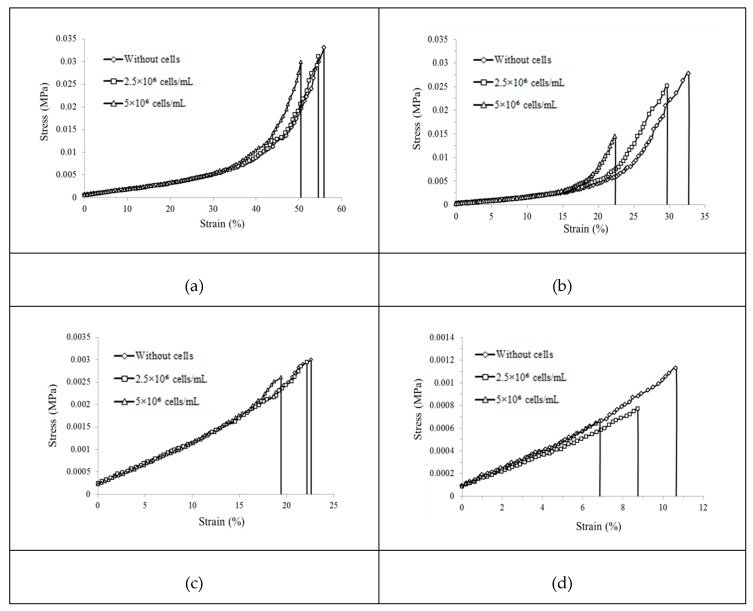
Stress–strain curves of the GelMA hydrogels with different GelMA concentrations at different incubation times: (**a**) 10% GelMA at 0 h, (**b**) 10% GelMA at 96 h, (**c**) 5% GelMA at 0 h, and (**d**) 5% GelMA at 96 h (a diamond represents without cells, a square represents 2.5 × 10^6^ cells/mL, and a triangle represents 5 × 10^6^ cells/mL).

Table 1 lists the summarized results of the mechanical testing of the cell-encapsulated GelMA samples. Figure 3 shows the effects of cell density on the mechanical properties of the 5% and 10% GelMA hydrogels. It can be seen that both the tensile strain and strength decreased with an increase in the cell density, but differences were very small for the 10% GelMA at 0 h incubation. Similar observations are also shown for the 5% GelMA at 0 h incubation. However, after 96 h of incubation, both the tensile strain and strength decreased significantly with an increase in the cell density. For the 10% GelMA hydrogels after 96 h of incubation, the maximum strain at failure decreased significantly from 33% to 23% when the cell density increased from 0 to 5 × 10^6^ cells/mL. Similarly, the tensile strength decreased significantly from 0.028 to 0.015 MPa when the cell density increased from 0 to 5 × 10^6^ cells/mL.

**Figure 4:**
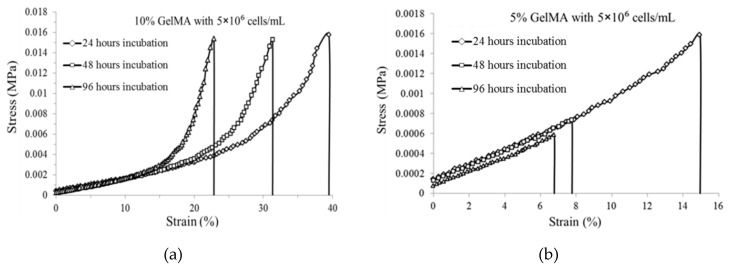
Stress–strain curves of GelMA (5 × 106 cells/mL) for (**a**) 10% GelMA and (**b**) 5% GelMA after 24 h, 48 h, and 96 h of incubation (a diamond represents 24 h of incubation, a square represents 48 h of incubation, and a triangle represents 96 h of incubation).

Figure 4 shows the effects of incubation time on the tensile strain and strength of the GelMA hydrogels with a cell density of 5 × 10^6^ cells/mL. For the 10% GelMA, the maximum strain at failure decreased significantly from 39% to 22%, and the tensile strengths slightly decreased from 0.015 MPa to 0.0145 MPa when the incubation time increased from 24 h to 96 h. For the 5% GelMA, with an increase in the incubation time, the maximum strain at failure decreased significantly from 13% to 7%, and the tensile strength also decreased significantly from 0.0013 MPa to 0.0006 MPa. The main reason for the decreased tensile strain and stress was that the encapsulated cells released enzymes and the enzymes cleaved the GelMA crosslinks. This cell-mediated enzymatic degradation significantly affected the tensile strain and strengths of the cell-encapsulated GelMA hydrogels. It was noted that the 10% GelMA had much higher tensile strain and strength than did the 5% GelMA. For example, after 96 h of incubation, the 10% GelMA had a maximum strain of 22% and a tensile strength of 0.0145 MPa, while the 5% GelMA only had a maximum strain of 7% and a tensile strength of 0.0006 MPa.

**Figure 5:**
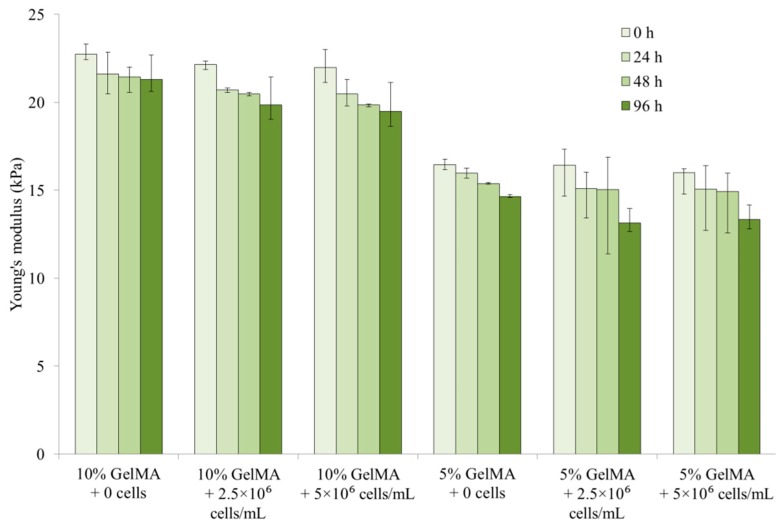
Tensile moduli of GelMA hydrogels with different GelMA concentrations and cell densities at different incubation times. The error bars indicate the minimum and maximum values of the calculated tensile moduli

Figure 5 shows the tensile moduli of the GelMA hydrogels with different GelMA concentrations and cell densities at different incubation times. It can be seen in Figure 5 that the tensile moduli of the GelMA hydrogels decreased slightly with an increase in the cell densities. For example, at 0 h incubation, the tensile moduli were 16.5 kPa for the 5% GelMA without cells, 16.4 kPa for the 5% GelMA with a cell density of 2.5 × 10^6^ cells/mL, and 16 kPa for the 5% GelMA with a cell density of 5 × 10^6^ cells/mL. When the cells were encapsulated within the GelMA hydrogels, the original GelMA parts were replaced by cells. The cell tensile modulus (1.5 kPa for 3T3 fibroblasts [30]) was much smaller than the GelMA tensile modulus. Hence, the GelMA hydrogels had decreased tensile moduli with an increase in cell density. However, in this study, the maximum cell concentration was 5 × 10^6^ cells/mL, and the associated cell volume fraction was only 0.88%. The effect of encapsulated cells on the tensile modulus was relatively small, resulting in the slight decrease. With the incubation time, the encapsulated cells started to release enzymes to cause GelMA hydrogel degradation. As the incubation time increased, the decrease in the GelMA tensile modulus due to an increase in the cell density became more and more pronounced. At 0 h incubation, the tensile moduli of the 5% GelMA decreased from 16.4 kPa to 16 kPa as the cell density increased from 0 to 5 × 10^6^ cells/mL, while at 96 h incubation, the tensile moduli of the 5% GelMA decreased from 14.7 kPa to 13.3 kPa as the cell density increased from 0 to 5 × 10^6^ cells/mL. Mauck et al. [31] reported similar conclusions, where the elastic moduli of hydrogels with different cell densities were similar under free and unconstrained incubation and swelling conditions, which was the case in our study. It is noted that an increase in cell density can cause a reduction in stiffness and strength, especially when the cells proliferate through the hydrogel network [31].

### 2.3. Physical Properties

**Figure 6:**
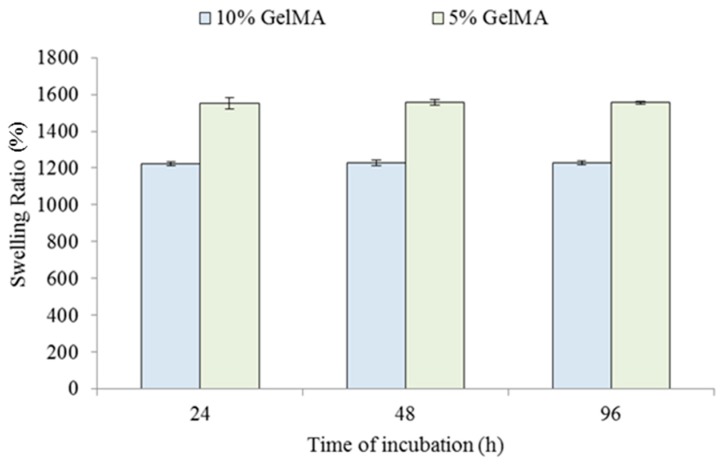
Swelling ratios with an increase in incubation time for 5% and 10% GelMA hydrogels. The error bars indicate the standard deviation

Swelling is an indicator of the amount of water sorption into a hydrogel [12]. In 3D bioprinting applications, cells are typically encapsulated within the hydrogels. The hydrogel swelling behaviors have direct effects on the nutrient retention in a hydrogel and waste egestion from cells out of a hydrogel. The study of swelling was performed on GelMA hydrogels with two different concentrations of 5% and 10%. The specimens were cultured in DPBS for 24, 48, and 96 h. Figure 6 shows the GelMA swelling ratio under different incubations times. It was seen that for both 5% and 10% GelMA, the swelling mainly occurred within the first 24 h, and after 24 h of incubation, the swelling ratio was almost constant. The swelling ratio of the 5% GelMA was greater than that of the 10% GelMA. The swelling ratios after 96 h of incubation were 1553 ± 30 and 1223 ± 9 for the 5% GelMA and the 10% GelMA, respectively. Zhao et al. reported that the swelling ratios were 1476 ± 28 and 719 ± 24 for the 5% GelMA and the 10% GelMA, respectively [12]. Our results were greater than those reported by Zhao et al. [12]. The differences might have been due to different exposure times. In this study, the exposure time was 60 s, while Zhao et al. used 180 s [12].

**Figure 7:**
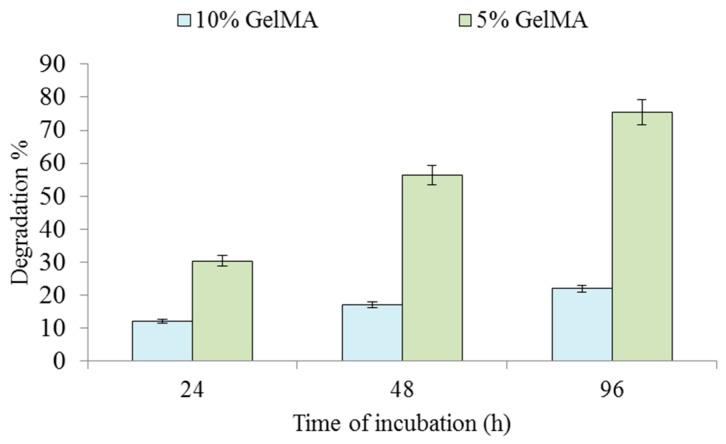
Degradation with an increase in incubation time for the cell-encapsulated GelMA hydrogels. The error bars indicate the standard deviation.

During the incubation period, the encapsulated cells release enzymes through their membranes. As these enzymes diffuse through the polymer network, they cleave existing crosslinks to cause polymer degradation. The specimens used for the degradation study in this paper contained 5% GelMA or 10% GelMA and a cell density of 5 × 10^6^ cells/mL. The specimens were incubated for 24, 48, and 96 h. Figure 7 shows the GelMA degradation percentage under different incubations times. It can be seen that as the incubation time increased, the GelMA degradation percentage increased. The 5% GelMA degradation was much faster than the 10% GelMA degradation. For the 5% GelMA, the degradation percentage increased significantly from 32% to 75% when the incubation time increased from 24 h to 96 h. In contrast, for the 10% GelMA, the degradation percentage only increased from 11% to 20% when the incubation time was from 24 h to 96 h. Zhao et al. [12] also investigated the degradation of 5% GelMA using the enzymes and reported the complete degradation of 5% GelMA after 72 h of incubation. There was a difference between the measured degradation percentage (75%) in this study and the result (100%) from Zhao et al. [12]. The difference was probably due to two reasons: 1) The collagenase solution used was refreshed every 2–3 days to maintain constant enzyme activity. The degradation of GelMA samples was uniform and steady. In contrast, the local cell density in our study may not have been uniform during incubation, considering cell migration and some dead cells in the center. 2) The encapsulated cells secreted their own ECM in the surrounding areas, which may have inhibited the diffusion of enzymes, resulting in a slower degradation. The degradation of hydrogels is facilitated by the hydrolysis-induced breaking of polymer chains and crosslinks due to enzymes secreted by cells [6]. The proliferation of cells and their subsequent secretion of enzymes are facilitated better in hydrogels such as GelMA with lower concentrations due to a reduction in the number of peptides to be cleaved [32]. Moreover, GelMA hydrogels with lower concentrations offer more space for encapsulated cells to extend outwards. GelMA hydrogels with higher concentrations are denser, which inhibits cell spreading. Hence, 5% GelMA hydrogels degrade faster than 10% GelMA hydrogels, which is consistent with the reported results [33]. It is noted that encapsulated cells proliferate to increase cell density within GelMA hydrogels with incubation time, which has been observed and reported by studies in the literature [5,13]. Moreover, the diffusion of enzymes that causes GelMA degradation slows down due to the new extracellular matrix.

### 2.4. Microstructure

The presence of pores in hydrogels is of great importance to the effective transfer of oxygen and nutrients to encapsulated cells [9]. These encapsulated cells are typically inhibited in their movement due to being surrounded by the hydrogel matrix and are reliant on the pores for the uptake of nutrients [10]. Pores of the hydrogels also provide space for cell attachment and proliferation [11]. During incubation, cell-mediated enzymatic degradation results in increased pore size [34]. This section systematically investigates the effects of the encapsulated cells on the GelMA pore size. The specimens used in this section contained 10% GelMA and different cell densities (0 and 5 × 10^6^ cells/mL).

**Figure 8:**
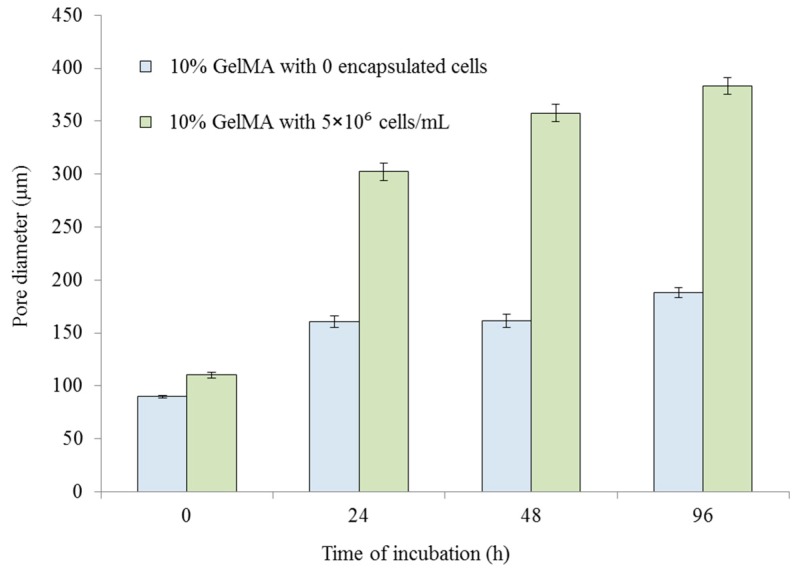
Pore sizes with an increase in incubation time for the cell-encapsulated GelMA hydrogels. The error bars indicate the standard deviation.

**Figure 9:**
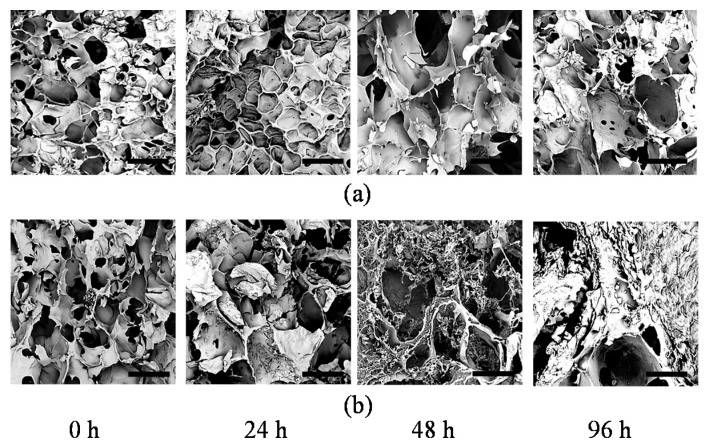
SEM images showing pore sizes after different hours of incubation for (**a**) 10% GelMA with no encapsulated cells and (**b**) 10% GelMA with a cell density of 5 × 10^6^ cells/mL (scale bar represents 300 µm).

Figure 8 shows the pore size of the 10% GelMA with different cell densities at different incubation times. It is seen that for the 10% GelMA without cells, the pore size increased significantly from 90 to 161 µm with an incubation from 0 to 24 h. However, from 24 h to 96 h, the pore size did not change significantly. The significant increase in the pore size in the first 24 h was mainly due to GelMA hydrogel swelling, which also occurred mainly within the first 24 h (Section 2.3). After 24 h, GelMA hydrogel swelling had no significant change, resulting in no significant change in the pore size. However, for the 10% GelMA with a cell density of 5 × 10^6^ cells/mL, the pore size increased with an increase in the incubation time. Within the first 24 h, the pore size increased significantly from 110 to 302 µm, which was due to both GelMA hydrogel swelling and cell-mediated enzymatic degradation. After 24 h, the pore size continued to increase, which was mainly due to cell-mediated enzymatic degradation. The increase in the pore size became slower compared to the first 24 h. In Figure 9, it is seen that the encapsulated cells significantly affected the pore size of the GelMA hydrogels. At 0 h, the GelMA hydrogels with and without cells had very similar pore sizes. After 96 h of incubation, the GelMA hydrogel without cells had a pore size of 187.9 ± 4.7 µm, while the GelMA hydrogel with cells had a pore size of 383.3 ± 7.8 µm. It is concluded that the encapsulated cells resulted in a larger pore size of the GelMA hydrogels. This observation was consistent with the reported conclusion using enzymes [34].

### 2.5. Cell Viability

**Figure 10:**
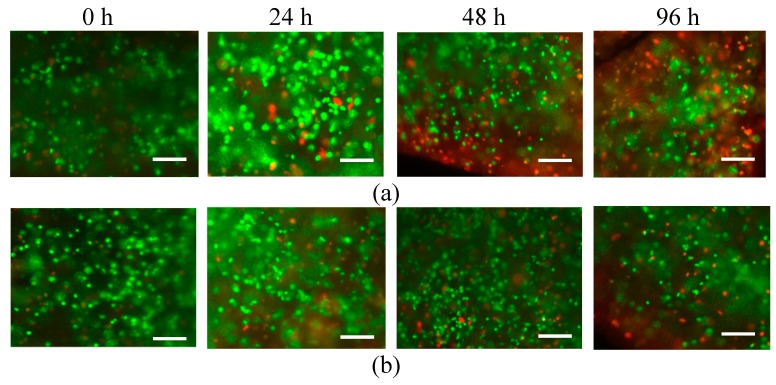
Postfabrication cell viability images after incubation for (**a**) 10% GelMA with 5 × 10^6^ cells/mL and (**b**) 5% GelMA with 5 × 10^6^ cells/mL (scale bar represents 300 µm).

The cell viability of the GelMA constructs was monitored throughout the experiment in order to verify the continued survival and proliferation of cells during incubation. The bioink used in this section contained 5% or 10% GelMA and a cell density of 5 × 10^6^ cells/mL. Then, the bioink was crosslinked under UV radiation to make disk specimens with a diameter of 12 mm and a thickness of 1 mm. The crosslinking conditions were a UV intensity of 6.9 mW/cm^2^ and an exposure time of 60 s. Figure 10 shows that for the bioink with 10% GelMA, the cell viability was measured at 85% after 24 h of incubation, 70% after 48 h of incubation, and 60% after 96 h of incubation. For the bioink with 5% GelMA, the cell viability was measured to be 90% after 24 h of incubation, 80% after 48 h of incubation, and around 70% after 96 h of incubation. The results show that most cells were alive during the incubation. Higher concentrations of GelMA consist of more abundant covalent bonds, which can lead to high rigidity and low porosity, significantly lowering cell viability [35]. It is noted in Figure 10 that the cell viability decreased with the incubation time. The main reason was that the 1-mm thickness of the specimens was relatively large for cell encapsulation applications. However, this thickness was chosen in this study because it benefits the shape fidelity of the specimens. If the thickness of the specimens is too small, the specimens cannot keep their shape after 96 h of incubation, and it is extremely difficult to fix the specimens into the tensile testing machine.

## 3. Materials and Methods

### 3.1. Materials

GelMA was synthesized as follows. Type A gelatin was dissolved in Dulbecco’s phosphate-buffered saline (DPBS) at 50 °C to prepare the 10% (*w/v*) gelatin solution. Then, 8% (*v/v*) methacrylic anhydride was added at a flow rate of 0.5 mL/min with the temperature kept constant at 50 °C. Two hours were allowed for reaction under constant stirring to induce methacrylation. The methacrylation reaction was stopped by adding warm DPBS to dilute the solution by 5X. The resulting solution was subject to dialysis for 1 week at 40 °C using a 12–14-kDa cutoff dialysis tubing to remove the methacrylic acid and other salts. Finally, lyophilization was performed on the solution for 1 week. The resulting white porous foam of GelMA was stored in a −80 °C freezer until further use. The concentrations of GelMA used in this study were 5% (*w/v*) and 10% (*w/v*). Irgacure 2959 (TCI America, Portland, OR, USA) was utilized as a photoinitiator at a concentration of 0.5% (*w/v*).

The model cells used for encapsulation in this study were NIH 3T3 mouse fibroblasts (ATCC, Rockville, MD, USA). Dulbecco’s Modified Eagle’s Medium (DMEM; Sigma-Aldrich, St. Louis, MO, USA) supplemented with 10% fetal bovine serum (FBS; HyClone, Logan, UT, USA) and 1% antibiotic/antimycotic solution (Corning, Manassas, VA, USA) was used to culture the fibroblasts in a humidified 5% CO_2_ incubator at 37 °C. The culture medium was replaced every 3 days. Cells were detached from their respective culture flasks by the addition of 0.25% Trypsin/EDTA (Sigma-Aldrich, St. Louis, MO, USA) at 37 °C for 2 min. The resulting cell suspension was subject to centrifuging at 1000 rpm for 5 min to separate the cell as a cell pellet, which was then resuspended in the GelMA solution at different cell concentrations. In this paper, we primarily selected three input factors with different levels: 1) the concentration of the GelMA precursor solution had two levels of 5% (*w/v*) and 10% (*w/v*); 2) the cell density had three levels of 0 cells, 2.5 × 10^6^ cells/mL, and 5 × 10^6^ cells/mL; and 3) the incubation time of cell-encapsulated constructs had four levels of 0 h, 24 h, 48 h, and 96 h. There were 24 different conditions, and each condition was repeated three times. Overall, 72 experiments were performed. The outputs of this study were the physical–mechanical properties and microstructures of GelMA hydrogels, including the ultimate tensile stress, the maximum strain at failure, the tensile modulus, the swelling ratio, the degradation percentage, and the pore size.

### 3.2. Methods

#### 3.2.1. Mechanical Property Measurement

Mechanical properties were characterized in terms of tensile modulus and strength. Bioinks with various concentrations of GelMA and different cell densities were crosslinked under UV radiation to make dog-bone-shaped specimens with encapsulated cells. The crosslinking conditions were a UV intensity of 6.9 mW/cm^2^ and an exposure time of 60 s. It is noted that there are potential issues observed with the tensile testing of hydrogels, such as the clamping of soft hydrogels and the adhesion of hydrogel samples to the clamps [36]. In this study, we utilized samples with a relatively large thickness of 1 mm to facilitate better gripping. The specimens were cultured in DMEM in a humidified 5% CO_2_ incubator at 37 °C for 24 h, 48 h, and 96 h. Then, the specimens were subject to uniaxial stretching at a constant rate of 1 mm/min until failure on a Dynamic Mechanical Analyzer (DMA Q800, TA Instruments, New Castle, DE, USA). Vice grips were used to grip the dog-bone structures during tensile testing. The corresponding stress and strain relation was obtained. The temperature was maintained at 37 °C during measurement.

#### 3.2.2. Physical Property Measurement

Swelling represents the amount of excess water that can be held within a hydrogel. The amount of water in a hydrogel can directly impact the permeation of nutrients into the gel and the egestion of cellular waste products out of the gel, as well as the shape fidelity of the printed constructs [5]. Hence, it was important to assess the swelling behaviors of the GelMA hydrogels. The bioinks with various concentrations of GelMA were crosslinked under UV radiation to make disk specimens with a diameter of 12 mm and a thickness of 1 mm. The crosslinking conditions were a UV intensity of 6.9 mW/cm^2^ and an exposure time of 60 s. Then, the specimens were incubated in DPBS in a humidified 5% CO_2_ incubator at 37 °C for up to 96 h. The specimens were wiped to drain excess water and weighted to obtain the swollen weight. Then, the specimens were subject to lyophilization at −50 °C for 12 h, and the corresponding dry weight of the specimens was measured. The swelling ratio was calculated using the following equation:

(1) $$S=\;\left(\frac{W_{s}-W_{d}}{W_{d}}\right)\times 100\%$$

where $W_{s}$ is the swollen weight of the specimens, and $W_{d}$ is the dry weight of the specimens. The swelling test was performed after 24 h, 48 h, and 96 h to study the swelling behaviors of the GelMA hydrogels with the incubation time. It is noted that the samples used for the swelling analysis had no encapsulated cells.

During the incubation of cellular constructs, encapsulated cells release enzymes through their membranes. As these enzymes diffuse through the polymer network, they act as catalysts for polymer degradation. Conventional approaches to studying degradation involve the incubation of hydrogel samples in a collagenase solution [5,12]. However, the observation of cell-mediated enzymatic degradation using cell-encapsulated specimens may allow for a more realistic analysis. The bioinks with 5% and 10% GelMA and a cell density of 5 × 10^6^ cells/mL were crosslinked under UV radiation to make disk specimens with a diameter of 12 mm and a thickness of 1 mm. The crosslinking conditions were a UV intensity of 6.9 mW/cm^2^ and an exposure time of 60 s. Then, the specimens were incubated in DMEM in a humidified 5% CO_2_ incubator at 37 °C for up to 96 h. The specimens were subject to lyophilization at −50 °C for 12 h and weighed to obtain the dry weight of the specimens. The degradation percentage was calculated using the following equation:

(2) $$D=\left(\frac{W_{d}-W_{t}}{W_{d}}\right)\times 100\%$$

where $W_{d}$ is the dry weight of the specimen immediately after fabrication, and $W_{t}$ is the dry weight of the specimen after a specific incubation time. The GelMA degradation was measured after incubations of 24 h, 48 h, and 96 h.

#### 3.2.3. Pore Size Measurement

The microstructure of the GelMA hydrogels was characterized in terms of pore size. The bioinks with a GelMA concentration of 10% and a cell density of 5 × 10^6^ cells/mL were crosslinked under UV radiation to make disk specimens with a diameter of 12 mm and a thickness of 1 mm. The crosslinking conditions were a UV intensity of 6.9 mW/cm^2^ and an exposure time of 60 s. The specimens were cultured in DMEM in a humidified 5% CO_2_ incubator at 37 °C for 24 h, 48 h, and 96 h. The specimens were subject to lyophilization at −50 °C for 12 h in order to preserve the microstructure of the GelMA hydrogels and viewed under a scanning electron microscope (ProX, Phenom, Phoenix, AZ, USA). The pore size was subsequently measured.

#### 3.2.4. Cell Viability Assessment

Cell viability was assessed using a fluorescent live/dead assay (Biotium, Fremont, CA, USA). Calcein acetoxymethyl ester (calcein AM) was used to label the live cells with a green fluorescent stain, and ethidium homodimer III was used to label dead cells with a green fluorescent stain. The samples were stained with the assay and subjected to incubation for 20 min at 37 °C in a humidified 5% CO_2_ incubator. Fluorescence images were captured with a fluorescence microscope (EVOS FL, Thermo Fisher Scientific, Waltham, MA, USA). The live and dead cells were quantified from the fluorescent images using the image processing software imageJ.

## 4. Conclusions

This paper studied the effects of encapsulated cells on the physical–mechanical properties and microstructure of cell-encapsulated GelMA hydrogels. The main conclusions are as follows: 1) With an increase in cell density, the maximum strain at failure and the ultimate tensile strength decreased significantly, while the tensile modulus did not change significantly; 2) GelMA swelling occurred within the first 24 h, and after 24 h the GelMA swelling ratio had no significant change. With an increase in cell density, the GelMA degradation percentage increased. 3) With an increase in cell density, the GelMA pore size increased. Cell viability was monitored throughout the study and was found to indicate the continuous survival of the encapsulated cells. Ten percent GelMA was observed to retain a high tensile strength and maximum strain after incubation with high cell densities compared to 5% GelMA. Five percent GelMA was observed to have a swelling ratio significantly higher than 10% GelMA, and 5% GelMA was also observed to have significantly higher degradation than 10% GelMA. This helps with filling the missing gap on the effect of encapsulated cells on GelMA constructs and provides significant information about cell–material interaction that will be highly useful for future bioprinting researchers and for the development of viable tissue development models

## Figures and Tables

**Table 1:** Summarized results of mechanical testing of cell-encapsulated GelMA samples.

GelMA Concentration (*w/v*)	Cell Density (× 10^6^ cells/mL)	Maximum Strain (%) at 0 h	Tensile Strength (MPa) at 0 h	Maximum Strain (%) at 96 h	Tensile Strength (MPa) at 96 h
5%	0	23	0.028	11	0.001
	2.5	22	0.027	9	0.0007
	5	19	0.026	7	0.0006
10%	0	57	0.033	33	0.028
	2.5	55	0.032	29	0.026
	5	51	0.030	23	0.015

## Abbreviations

DMEM	Dulbecco’s Modified Eagle’s Medium
DPBS	Dulbecco’s phosphate-buffered saline
ECM	Extracellular matrix
GelMA	Gelatin methacrylate
UV	Ultraviolet
